# Resveratrol alleviates chemotherapy-induced oogonial stem cell apoptosis and ovarian aging in mice

**DOI:** 10.18632/aging.101808

**Published:** 2019-02-14

**Authors:** Meng Wu, Lingwei Ma, Liru Xue, Wenlei Ye, Zhiyong Lu, Xiang Li, Yan Jin, Xian Qin, Dan Chen, Weicheng Tang, Yingying Chen, Zixin Hong, Jinjin Zhang, Aiyue Luo, Shixuan Wang

**Affiliations:** ^1^Department of Obstetrics and Gynecology, Tongji Hospital, Tongji Medical College, Huazhong University of Science and Technology, Wuhan, Hubei 430030, China; ^2^Hubei Key Laboratory of Embryonic Stem Cell Research, Tai-He Hospital, Hubei University of Medicine, Shiyan, Hubei 442000, China; ^*^Equal contribution

**Keywords:** resveratrol, chemotherapy, ovarian aging, oogonial stem cell, apoptosis

## Abstract

Chemotherapy-induced ovarian aging not only increases the risk for early menopause-related complications but also results in infertility in young female cancer survivors. Oogonial stem cells have the ability to generate new oocytes and thus provide new opportunities for treating ovarian aging and female infertility. Resveratrol (3,5,4′-trihydroxy-trans-stilbene) is a natural phenol derived from plants, that has been shown to have positive effects on longevity and redox flow in lipid metabolism and a preventive function against certain tumors. To evaluate whether resveratrol could promote the repair of oogonial stem cells damage in a busulfan/cyclophosphamide (Bu/Cy)-induced accelerated ovarian aging model, female mice were administered 30 and 100 mg/kg/d resveratrol through a gavage for 2 weeks. We demonstrated that resveratrol (30 mg/kg/d) relieved oogonial stem cells loss and showed an attenuating effect on Bu/Cy-induced oxidative apoptosis in mouse ovaries, which may be attributed to the attenuation of oxidative levels in ovaries. Additionally, we also showed that Res exerted a dose-dependent effect on oogonial stem cells and attenuated H_2_O_2_-induced cytotoxicity and oxidative stress injury by activating Nrf2 *in vitro*. Therefore, resveratrol could be of a potential therapeutic drug used to prevent chemotherapy-induced ovarian aging.

## INTRODUCTION

Cancer is one of the most important global public health problems worldwide. Thousands of young women are diagnosed with cancer every year and exposed to cytotoxic chemotherapy regimens and radiation, which have a substantial negative impact on reproduction [1]. These treatments may cause infertility and ovarian aging by inducing genomic damage and apoptotic death of oocytes [2]. Therefore, the preservation of ovarian function and fertility has become one of the major quality of life issues for cancer survivors. Currently available fertility preservation strategies such as cryopreservation of gametes and ovarian tissue can help women achieve pregnancy. Nevertheless, these strategies cannot reverse menopause or restore ovarian function [3]. Therefore, any drugs that preserve the function of ovaries during chemotherapy are urgently needed.

Ovarian aging is characterized by impaired ovarian reserve, namely, a gradual decline in oocyte quality and the quantity of oocytes in ovaries. It is typically speculated that the depletion of the primordial follicular pool leads to ovarian aging [4, 5]. However, in recent years, some researchers have indicated that the physiological condition of oogonial stem cells (OSCs) directly determines ovarian reserve function [6]. The significance of OSCs is mainly attributed to the ability to regenerate the postnatal follicle pool in juvenile and adult ovaries [6–8]. Furthermore, a recent study suggested that chemotherapy may lead to apoptosis and dysfunction of OSCs, which may be caused by a disorder of homeostasis of the ovary [8].

Resveratrol (3,5,4’-trihydroxy-trans-stilbene, Res), a plant-derived natural compound, has a wide variety of biological properties, including its anti-inflammatory, cardioprotective, anti-cancer and anti-aging effects [9]. These effects are mainly attributed to its antioxidant activity as a free radical scavenger [10]. Res has been shown to mimic the beneficial health outcomes of caloric restriction [11]. Moreover, Res is also the only acknowledged nonpharmacological intervention capable of slowing the aging process [12] and increasing lifespan of organisms including yeast, nematodes, flies and mammals [13]. Previous studies have addressed Res and its effects on the proliferation, senescence and differentiation of several types of stem cells. Res has been reported to relieve H_2_O_2_-induced cellular senescence in MSCs by activating SIRT1 [14]. Additionally, a moderate concentration of Res has been shown to exert protective effects on X-ray-treated and ethanol-induced embryonic stem cells (ESCs) without damaging their genomic stability [15, 16]. In addition, Res showed a dose-dependent effect on spermatogonial stem cells (SSCs) and rescued SSC loss in a busulfan-induced infertile mouse [17].

Although the mechanism of chemotherapy toxicity on ovaries has not been clearly explained to date, it is speculated to increase ROS production and decrease antioxidant activity because of chemotherapy-induced oxidative stress [18]. Resveratrol has antioxidant properties that can strengthen endogenous cellular antioxidant systems that have direct ROS scavenging properties. We hypothesized that Res also has the ability to restore ovarian function. Therefore, in this study, we demonstrated that Res protected OSC biological activity and thus preserved ovarian function in Bu/Cy-induced mouse ovary aging models. In addition, we identified the biological and genetic characteristics of *in vitro* cultured mouse OSCs and then assessed the effects on OSC viability, proliferation and apoptosis.

## RESULTS

### Res improved ovarian aging induced by chemotherapy

**Figure 1:**
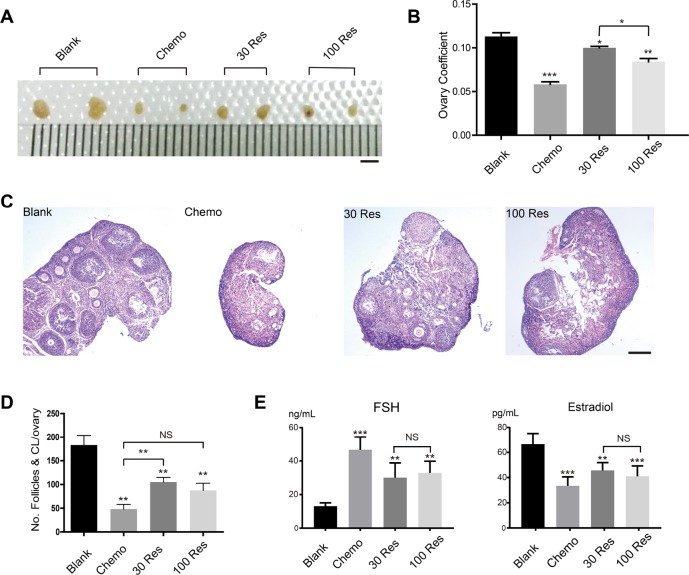
**Res improved ovarian aging induced by chemotherapy**. (**A**) Bright field images of ovaries from 4 different groups. Scale bar, 2 mm. (**B**) The ovary coefficient of the 4 groups. (**C**) Representative images of HE stained of ovaries from the 4 groups to analyze the effects of Res on mouse infertility. Scale bar: 200 μm. (**D**) The number of follicles and corpus luteum in each ovary of the 4 groups. (**E**) Analysis of the hormone levels of FSH and Estradiol from the 4 groups.

The dose of Res ranged from 24 to 400 mg/kg/d when it was reported to act as an anti-aging therapy [19, 20]. In our study, Res was administered by gastrogavage at a low dosage of 30 mg/kg/d (30 Res group) and a high dosage of 100 mg/kg/d (100 Res group) to interfere with infertility mice treated with busulfan/cyclophosphamide (Bu/Cy). The results showed that the ovaries were seriously damaged by the Bu/Cy treatment (reduced volume and oocyte loss). However, after treatment with Res, especially in the 30 Res group, the morphology and weight of the ovaries were recovered compared with the chemotherapy group (Chemo group) (Figure 1A, 1B). In addition, the hematoxylin and eosin-stained tissue showed that the number of follicles was increased in the 30 Res group (Figure 1C); however, there was no significant difference between the 100 Res group and Chemo group (Figure 1D). Additionally, the levels of the sex hormones 17β-estradiol (E2) and follicle-stimulating hormone (FSH) changed, and an increase in E2 and a decrease in FSH were observed in the 30 Res and 100 Res groups compared with the Chemo group (Figure 1E). Collectively, we concluded that the ovarian function of the 30 Res group recovered after treatment with chemotherapy. The hormone level of the 30 Res group was elevated; however, there was no significant difference in hormone levels between the 30 Res group and 100 Res group.

### Resveratrol improved the renewal ability of OSCs in chemotherapy mice

**Figure 2:**
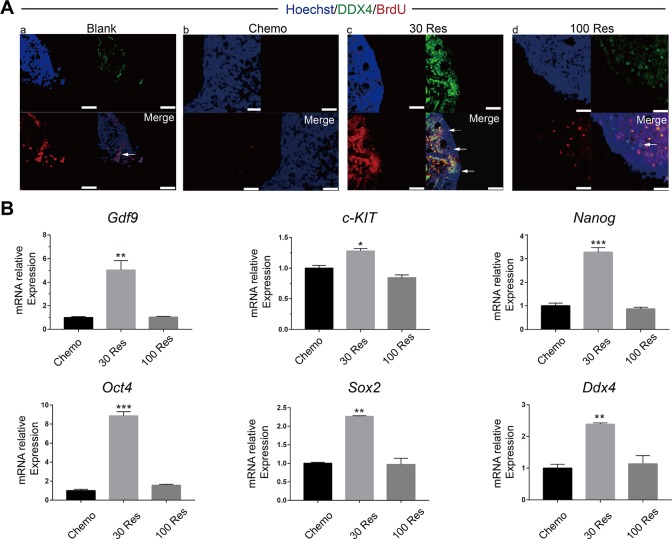
**Resveratrol improved renewal capacity of OSCs in chemotherapy mice**. (**A**) Dual immunostaining for BrdU (red) and DDX4 (green) of OSCs (arrow) were observed near the surface epithelium of mouse ovaries in the (a) Blank, (b) Chemo, (c) 30 Res and (d) 100 Res groups. Scale bar, 200 μm (**B**) Relative mRNA expression of *c-KIT*, *Oct4*, *Sox2*, *Nanog*, *Gdf9* and *Ddx4* in the different groups. *p < 0.05; **p < 0.005; ***p < 0.001.

To identify and confirm whether Res promoted the renewal of OSCs, morphological and histological analyses of 5’-bromodeoxyuridine (BrdU) and DDX4 protein double-positive cells were used to identify OSCs [21, 22]. The presence of BrdU–DDX4 double-positive cells near the ovarian surface epithelium was observed. The OSC pool decreased four weeks after chemotherapy. In Res treated mice, the number of OSCs per ovary increased and plateaued, and the 30 Res group showed better recovery compared with the 100 Res group (Figure 2A). In addition, we analyzed the mRNA expression levels of stemness- and germline-related genes (*c-KIT*, *Oct4*, *Sox2*, *Nanog*, *Gdf9* and *Ddx4*) by RT-PCR. Compared with the chemotherapy group, 30 Res significantly increased the expression levels of the related genes (p < 0.05) (Figure 2B), which suggested that Res alleviated the damage of chemotherapeutic drugs to OSCs. However, there was no improvement observed in the 100 Res group. Collectively, these results demonstrated that the low dose of Res (30 mg/kg) reduced the depletion of OSCs caused by chemotherapy.

### Resveratrol attenuated oxidative stress in ovaries induced by chemotherapy

**Figure 3:**
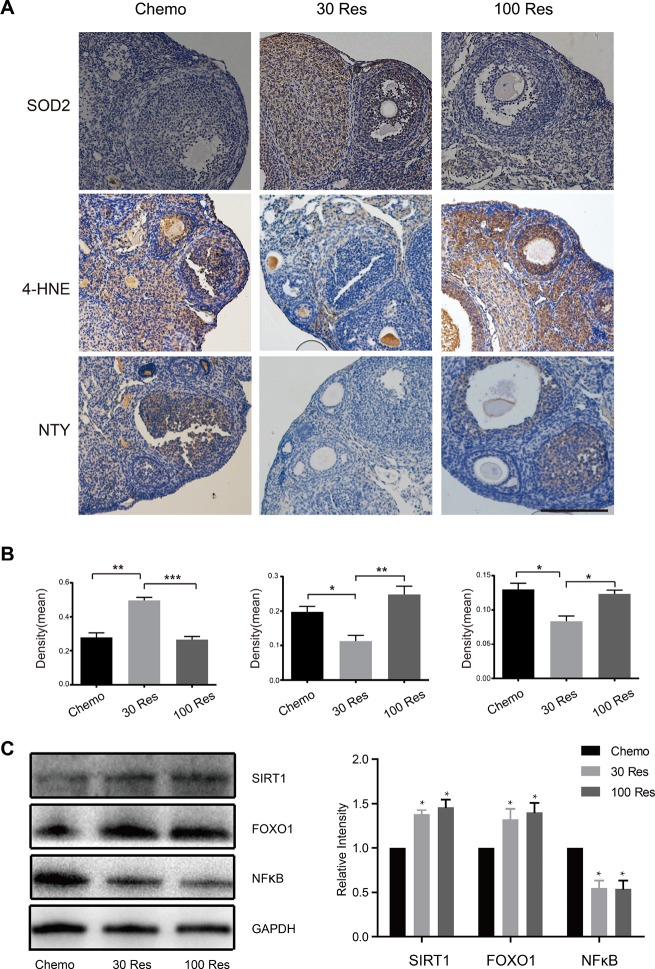
**Resveratrol attenuated the oxidative stress of ovaries induced by chemotherapy**. (**A**) The expression of SOD2, 4-HNE and NTY in the interstitial cells and follicles in the ovaries of Chemo, 30 Res and 100 Res groups of mice. Scale bar: 200 μm. *p < 0.05; **p < 0.005; ***p < 0.001. (**B**) The quantification of IHC. (**C**) The western blotting and quantification of of indicated proteins of ovaries in 3 groups. *p < 0.05; **p < 0.005; ***p < 0.001; NS: not significant.

Oxidative stress is accompanied by the pathological process of aging [23], and may promote ovarian aging [24]. Superoxide dismutase 2 (SOD2) is a free radical scavenger that plays an important role in protecting cells from the oxidative toxicity of ROS [25]. Nitrotyrosine (NTY) is a product of tyrosine nitration, commonly recognized as an indicator or marker of cell damage, inflammation and nitric oxide production [26]. 4-Hydroxynonenal (4-HNE) is generated by lipid peroxidation during the oxidation of lipids and might influence the cellular senescence process and contribute to organismal aging. These molecules are widely accepted as biomarkers of oxidative DNA, protein, and lipid damage in biological systems [27]. In our study, SOD2, NTY and 4-HNE were analyzed by immunohistochemistry. Compared with the Chemo group, the SOD2 level was increased in the 30 Res group (p < 0.05), while the oxidative damage markers (NTY and 4-HNE) were decreased (p < 0.05), but the high dosage of Res (100 mg/kg/d) did not have these effects (Figure 3A, 3B). Additionally, SIRT1 is a key factor in inhibiting the oxidative stress response, and resveratrol is a natural activator of SIRT1. The results of the Western blot showed that the expression levels of SIRT1 and FOXO1 were increased in the Res group, and the inflammatory factor NFkB was decreased in the Res group, indicating that Res may alleviate oxidative stress induced by chemotherapy by activating the SIRT1/FOXO1 pathway (Figure 3C, 3D). Overall, the results suggested that a low dose of Res (30 mg/kg/d) reduced the oxidative damage, while enhancing the antioxidant activity in aging female mice.

### Characterization of established mouse OSCs

**Figure 4:**
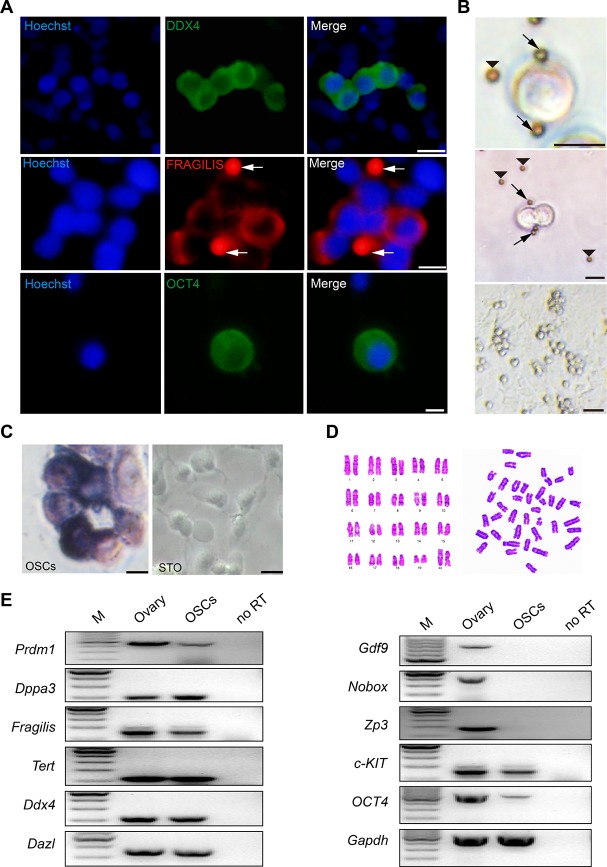
**Morphology and characteristics of OSCs**. (**A**) Immunofluorescence of DDX4 (green), FRAGILIS (red) and OCT4 (green) in mOSCs. Arrows: magnetic beads. Scale bars, 10 μm. (**B**) Examples of OSCs isolated with the Fragilis antibody and the associated pattern of OSCs with immunomagnetic beads. Arrows: magnetic beads associated with OSCs. Arrowheads: free beads. Scale bars, 10 μm, 10 μm, and 25 μm (From top to bottom). (**C**) Alkaline phosphatase staining for OSCs and STO. Scale bars, 10 μm. (**D**) Cytogenetic analysis showed that OSCs possessed a normal karyotype (40, XX). (**E**) Reverse transcription PCR analysis for the expression profile of OSCs using ovarian tissue as a positive control. M: 100 bp DNA marker; No RT, PCR of RNA sample without reverse transcriptase.

OSCs were isolated from 6-week-old mouse ovaries and sorted using MACS following a previously reported protocol [28]. After purification and several passages *in vitro* for approximately 1 month, we observed the morphology of OSCs, which indicated a group string with a large ratio of nuclear plasma (Figure 4A). To confirm the identity of OSCs, we performed immunofluorescence analysis of the isolated OSCs, which indicated that DDX4, OCT4 and FRAGILIS were expressed in OSCs (Figure 4B). In addition, OSCs showed positive staining for alkaline phosphatase (AP) compared to the negative control STO (Figure 4C). Furthermore, a cytogenetic analysis showed that the cells had a normal karyotype (40, XX) (Figure 4D). Finally, PCR analysis showed that OSCs expressed the germ cell genes *Prdm1*, *Dppa3*, *Fragilis*,* Tert*, *Ddx4* and *Dazl*, and the specific genes for oocytes including *Gdf9*, *Nobox* and *Zp3* were not expressed in OSCs. The genes involved in stem cell self-renewal (*c-KIT*, *Oct4*) also showed positive results (Figure 4E). Collectively, the results suggest that cultured OSCs are chromosomally normal and maintain the characteristics of germline stem cells.

### The effects of resveratrol on OSCs were dose dependent

**Figure 5:**
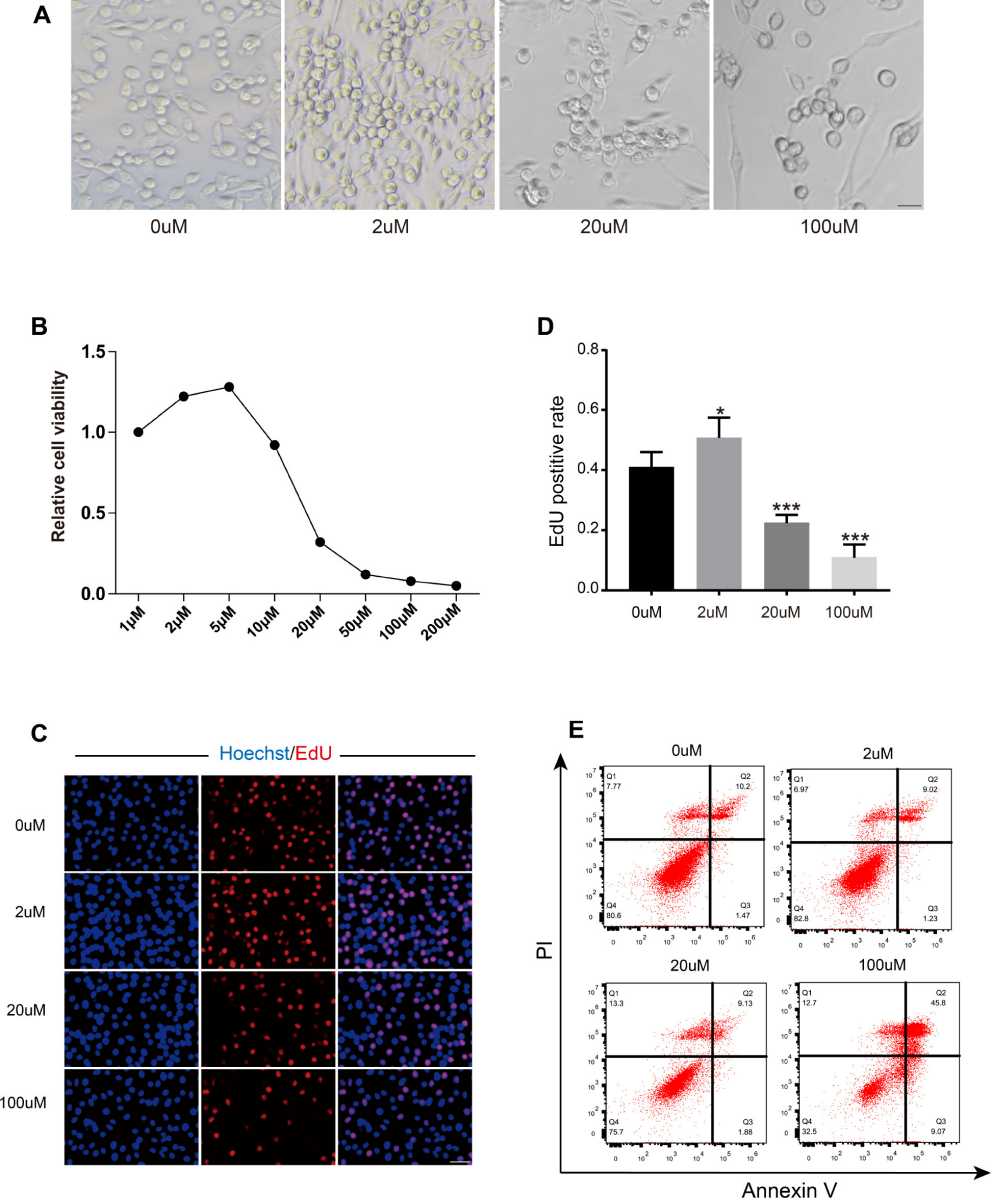
**Resveratrol had a dose-dependent effect on OSCs**. (**A**) The morphology of OSCs after treatment with Res at different concentrations. Scale bar, 20 μm. (**B**) Cell viability of OSCs after treatment with Res. (**C**) Immunofluorescence images of EdU-positive cells. Scale bar: 50 μm. (**D**) The EdU-positive rate of OSCs after treatment with Res. *p < 0.05; **p < 0.005; ***p < 0.001; NS: not significant. (**E**) Flow cytometry apoptotic analysis of OSCs after treatment with gradient concentrations of Res.

A concentration of Res between 1-1000 μM has been shown to have antioxidant biological properties in cells *in vivo* [29]. In human breast cancer cells (MCF7 cells), 10-150 μM Res negatively regulated breast cancer cell growth via Bcl-2 and NFkB [30]. Res (500 μM) also stimulated SIR2, thus increasing DNA stability and extending the lifespan in *Saccharomyces cerevisiae* [31], indicating that Res could exert biological properties through a wide range of concentrations. After we obtained the OSC lines, we further explored the effects of Res on OSCs *in vitro*. OSCs were observed as typical colonies in the presence of low-dose Res. The number of colonies was significantly decreased in the high-dose group (Figure 5A). To further test the effects of Res on OSCs, we examined cell viability by using a CCK-8 kit. The results showed that low concentrations of Res (2 and 5 μM) exerted a beneficial effect on cell viability, whereas the activity of OSCs was remarkably suppressed when exposed to high-dose Res (50, 100 and 200 μM) (Figure 5B). Next, we detected the EdU- positive OSCs to analyze the proliferation rate. The low concentration of Res (2 μM) promoted the proliferation of OSCs. Moreover, after 24 h incubation in high concentrations of Res (100 μM), DNA synthesis was more than 50 % inhibited compared to the control (Figure 5C, 5D). Additionally, the flow cytometry assay showed that the highest incidence of apoptosis reached 45.8 % with the 200 μM concentration of Res (Figure 5E). Overall, Res had a dose-dependent effect on OSCs *in vitro*, which was indicated by a noticeable inhibition in cell viability and proliferation with increasing doses of Res.

### Resveratrol attenuated H_2_O_2_-induced cytotoxicity and oxidative stress injury in OSCs

**Figure 6:**
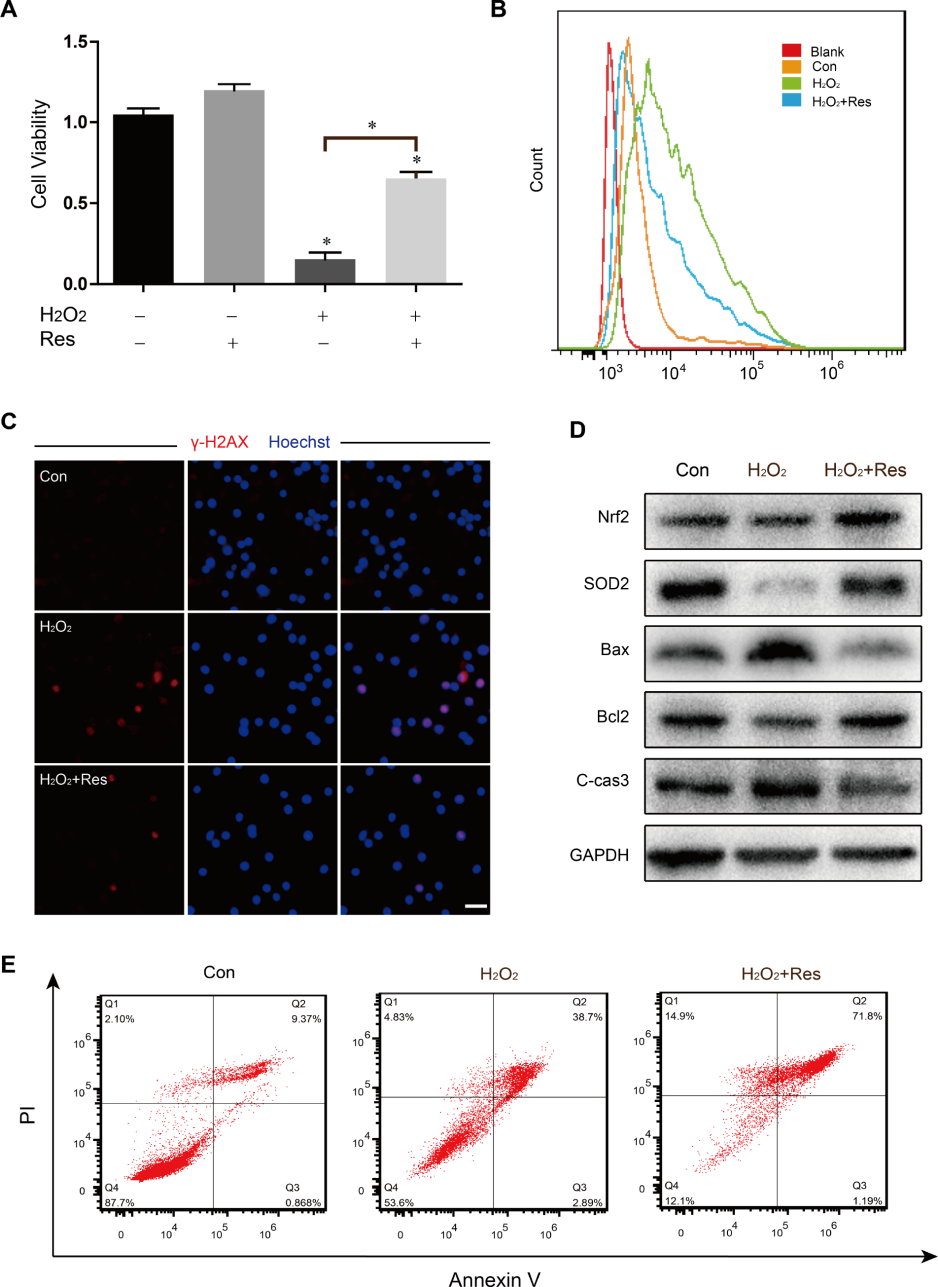
**Resveratrol attenuated H2O2-induced cytotoxicity and oxidant stress injury in OSCs**. (**A**) CCK8 assay for treated OSCs; *p < 0.05. (**B**) Analysis of intracellular ROS by cell flow cytometry. (**C**) Immunofluorescence staining of γ-H2AX and Hoechst. Scale bar: 50 μm. (**D**) Western blotting of related protein expression levels in treated OSCs. (**E**) The flow cytometry apoptotic analysis of treated OSCs.

Because oxidative damage plays a crucial role in chemotherapy-induced ovary aging and apoptosis of stem cells, to further assess the protective effect of Res against oxidative stress induced by Bu/Cy, however, which could not interfere directly with cells *in vitro*. Thus we employed H_2_O_2_, which can cause various types of cellular injuries as a type of ROS. Indeed, as shown in Figure 6A, OSCs treated with H_2_O_2_ showed a significant reduction in cell viability, which was prevented by Res pretreatment. To evaluate the level of oxidative stress in the cells, we examined the intracellular ROS level. Based on our results, H_2_O_2_ stimulation greatly increased ROS, while Res reduced ROS levels after H_2_O_2_ stimulation (Figure 6B), suggesting that resistance to H_2_O_2_ injury was improved by Res. Then we used immunofluorescence to detect the expression of γ-H2AX, which indicated the breakage of the double DNA strand, and the DNA damage induced by H_2_O_2_ was alleviated in the Res group (Figure 6C). In addition, the caspase cascade induces cell apoptosis after oxidative stress, and we observed that the apoptosis rate increased markedly after H_2_O_2_ stimulation and that Res treatment significantly reduced the level of cell apoptosis (Figure 6D). The expression levels of cleaved caspase-3 and Bax in OSCs also decreased after Res treatment, indicating that Res reduced cell apoptosis through inhibition of mitochondrial stress (Figure 6E). Nrf2 is a key antioxidant transcription factor that activates the adaptive response. SOD2 is a free radical scavenger which protects cells from oxidative toxicity. We analyzed the protein expression levels in the different groups to identify the degree of oxidative stress. The results showed that Res treatment promoted the expression of Nrf2 and SOD2, suggesting that Res protected against H_2_O_2_-induced cell death by decreasing oxidative stress (Figure 6E).

## DISCUSSION

Ovarian aging is a major and common long-term side effect of cancer chemotherapy. As representative alkylating agents, Bu/Cy may lead to ovarian aging by a variety of mechanisms involving acute ovarian vascular toxicity [32], cellular dysfunction of ROS [33] and severe depletion of the primordial follicle pool. Notably, an increased accumulation of intracellular ROS may promote the progression of ovarian aging, indicating that overproduction of ROS could decrease oocyte quality, development and maturation. ROS accumulation is highly reactive and toxic to mitochondrial DNA, thereby causing mitochondrial DNA mutations. Intriguingly, abnormal accumulation of mitochondrial DNA deletion may serve as a regulating factor of dysfunction in ovarian aging.

Increasing evidence suggests that dysregulated formation of ROS may promote premature senescence of stem cells and thus impede normal tissue homeostasis. The redox status of the stem cell microenvironment plays an essential role in stem cell maintenance. The niche that stem cells reside in is characterized by hypoxia and low ROS [34]. High levels of ROS could have detrimental effects on stem cells. However, physiological levels of ROS are required in the regulation of stem cell fate decisions [35]. The accumulation of ROS, which is associated with DNA damage and mitochondrial dysfunction, is a detrimental cycle that leads to OSCs dysfunction and apoptosis [36]. ROS activated apoptosis induction by mitochondria-dependent pathways [37]. As the epicenter of ROS management in stem cells, mitochondrial activity regulates ROS generation and influences the function and fate of stem cells. Categorically, busulfan could inhibit spermatogonia, leading to spermatogenic failure in the testis [38–40]. Further studies have shown that combined injection of Bu (30 mg/kg) and Cy (120 mg/kg) was sufficient to sterilize female mice and destroy the germline stem cell pool [21, 22].

According to previous evidence, Res has been ascertained to be a powerful ROS scavenger in mitochondria and could attenuate oxidative injury through the regulation of ROS homeostasis [41, 42]. Recent studies have suggested that Res could potentially delay the aging process by modulating the hallmarks of aging, including oxidative damage, inflammation, telomere attrition and cell senescence [43]. The ability of Res to improve mitochondrial function requiring SIRT1 is dose-dependent. Price et al. [44] demonstrated that mice treated with Res at a low dosage demonstrated increased mitochondrial function and biogenesis, AMPK activation and NAD^+^ levels in skeletal muscle. In addition, Res has shown a protective function against inflammation in a series of studies involved in the aging of the liver, brain and skin [45–47]. From the perspective of telomeres, Res could attenuate telomere dysfunction through by activating Werner syndrome ATP-dependent helicase (WRN helicase), which contributes to telomere maintenance [48]. Furthermore, it has been reported that Res provides protective effects to damaged stem cells, such as embryonic stem cells (ESCs) [49], embryonic neural stem cells [50], and SSCs [17], by maintaining genomic stability and accelerating recovery from DSBs (double-strand breaks) [16]. However, there has been little research about whether Res has protective functions in a chemotherapy-induced ovarian aging model and whether Res could exert protective effects on female germline stem cells from apoptosis. Our study showed that: 1) chemotherapy led to apoptosis and ovarian aging, which may have been due to oxidative stress injury; 2) Res alleviated chemotherapy-induced OSC apoptosis and aging; 3) Res exerted a dose-dependent effect on OSCs *in vitro*; and 4) Res attenuated H_2_O_2_-induced cytotoxicity and oxidative stress injury by acting Nrf2 and SOD2 in OSCs. To explore the recovery mechanisms of Res, we analyzed the oxidative stress level in the ovaries of the model mice (Figure 3). SOD2, which transforms toxic superoxide into hydrogen peroxide and diatomic oxygen, is a member of the superoxide dismutase family. For this reason, SOD2 functions as a scavenger of ROS to protect cells against apoptosis [51]. SOD2 was markedly increased in the 30 Res group compared with the Chemo group. Furthermore, the oxidative lipid damage marker 4-HNE and oxidative protein damage marker NTY showed a lower expression level in the 30 Res group. As a result, the alterations in these biomarkers showed that 30 mg/kg/d Res alleviated the oxidative stress level in ovaries subjected to chemotherapy. As free radical scavengers, a low concentration of Res (30 mg/kg/d) attenuated the oxidative stress level in chemotherapy ovaries, thus providing a suitable microenvironment for OSCs, which may protect stem cells from chemotherapy damage (Figure 6A). However, the protective effect of the high concentration of Res (100 mg/kg/d) was not observed; the reason may be that the high concentration of Res functions as a chemotherapeutic agent, which can induce apoptosis of liver and colon cancer cells by the mitochondria, p62, GSK3β and other pathways [52].

Oxidative stress is due to an imbalance between ROS production and antioxidant defenses. Exogenous antioxidants or the modulation of antioxidant enzymes can be expected to reduce oxidative stress. During chemotherapy, the damaged tissue produces excessive amounts of ROS, causing oxidative stress that results in mitochondrial oxidative phosphorylation, ATP depletion, increases of intracellular calcium, and activation of membrane phospholipid proteases [53]. Nrf2 and SOD2 are two major proteins that control intracellular oxidative stress [54]. As a natural antioxidant, previous studies have shown that Res can directly scavenge ROS, and exogenously administered Res activated the expression and activity of antioxidant enzymes such as SOD, glutathione peroxidase, and catalase through transcriptional regulation by Nrf2, activator protein 1, FOXO, and SP-1 or through enzymatic modification [55]. Our data are consistent with these studies, demonstrating that Res treatment dramatically upregulates Nrf2 and SOD2 expression *in vitro*.

However, some limitations could not be ignored in this study. The effects of Res on cellular growth and apoptosis are not a universal conclusion, which could be affected by different doses and different biological systems and the specific mechanisms need to be further explored. In addition, the results obtained from mouse models may not be applicable to humans. More studies are urgently needed to clarify the effects of Res on the human reproductive system. Additionally, the details about whether and how Res acted on OSCs so as to accomplish the rescuing effect remains exploration.

In summary, our study demonstrated that Res alleviated chemotherapy-induced oogonial stem cell apoptosis and ovarian aging *in vivo*. Additionally, Res had a dose-dependent effect on OSCs and attenuated H_2_O_2_-induced cytotoxicity and oxidative stress injury by activating Nrf2 and SOD2 *in vitro*. Although there are some limitations to our study, it might provide an efficient approach for therapeutic intervention to promote OSC biological activity and alleviate OSC loss in Bu/Cy-induced mice. From a clinical point of perspective, Res might help patients with primary ovarian insufficiency (POI) and cancer after chemotherapy to relieve gonadal toxicity, which is crucial for the future application of stem cells in regenerative medicine and the field of anti-aging research.

## MATERIALS AND METHODS

### Animal experimental protocol and ethics statement

Six-week-old healthy adult female C57BL/6 mice (weighing 19.63 ± 0.64 g) used in this study were obtained from the Center of Medical Experimental Animals of Hubei Province (Wuhan, China). The mice were housed in wire cages at a controlled temperature of 25 °C under a 12 h light-dark cycle and fed ad-libitum. The mice received a single intraperitoneal injection of busulfan (30 mg/kg) and cyclophosphamide (120 mg/kg) diluted in DMSO. After 2 weeks of the single injection of Bu/Cy treatment, Res (30 mg/kg/d and 100 mg/kg/d) was administrated by gastrogavage on alternating days for 2 weeks, and each group consisted of 15 mice. All mice were euthanized to collect their ovaries after the day of the last Res gavage treatment. Two hours before sacrifice, the mice were injected intraperitoneally with BrdU (Sigma-Aldrich, St. Louis, MO, USA) dissolved in saline (100 mg/kg) for a subsequent immunofluorescence assay. The blank group (Blank) consisted of mice treated with carboxymethylcellulose (CMC) by gavage as a vehicle control. The chemotherapy group (Chemo) consisted of mice that were treated with Bu/Cy and were administered 0.5 % CMC by gavage. The 30 Res group (30 Res) consisted of mice that were treated with Bu/Cy and were administered Res (30 mg/kg/d) dissolved in 0.5 % CMC by gavage. The 100 Res group (100 Res) consisted of mice that were treated with Bu/Cy and were administered Res (100 mg/kg/d) dissolved in 0.5 % CMC by gavage. All of the experimental procedures used for live animal care and handling in this study were approved by the ethics committee of Tongji Hospital, Tongji Medical College, Huazhong University of Science and Technology in China.

### Cells and cell experimental protocol

OSCs were isolated and established from 6-week-old female mice using protocols described previously [21, 28, 56]. The cells were digested and purified by MACS using a Fragilis antibody (ab15592, Abcam, Cambridge, MA, USA) and goat anti-rabbit IgG microbeads (130-048-602, Miltenyi Biotec, Auburn, CA, USA) after 2 or 3 d. The sorted cells were cultured on feeder cells with OSC culture medium, which consisted of α-MEM (Life Technologies, Waltham, MA, USA), 10 % fetal bovine serum (Gibco, Waltham, MA, USA), 1000 units/ml LIF (Millipore, Darmstadt, Hesse-Darmstadt, Germany), 1 ng/ml bFGF (BD Biosciences, San Diego, CA, USA), 10 ng/ml EGF (Sigma-Aldrich), 20 ng/ml human GDNF (R&D systems, Minneapolis, Minnesota, USA), 1 mM NEAA (Gibco), 1 mM sodium pyruvate (Gibco), 0.1 mM β-mercaptoethanol (Millipore), 1× concentrated N-2 supplement (R&D systems) and 1×concentrated penicillin-streptomycin. The medium was changed every 2-3 d. The cells were subcultured every 3-4 d and maintained at 37 °C in a humidified 5 % CO_2_ incubator. Resveratrol (Sigma-Aldrich) was dissolved in DMSO (PanReac AppliChem, Barcelona, Spain) for the *in vitro* studies. Cultured OSCs were incubated with 10 μM Res for 2 h, and then treated with 50 μM H_2_O_2_ in the culture medium. After 2 h, the medium was removed, and the cells were subjected to the subsequent experiments. (Sigma-Aldrich, St. Louis, MO, USA).

### Cell viability assay

OSCs were cultured in 96-well plates and treated with a gradient concentration of Res (0- 200 μM). After 24 h of treatment, 100 μl CCK8 (Dojindo, Tokyo, Japan) solution per well was added and the plate was incubated for 3 h in a 37 °C incubator. Then the absorbance of each well was measured at a wavelength of 450 nm using a microplate spectrophotometer.

### Immunofluorescence

The ovaries were fixed in 4 % paraformaldehyde (PFA) overnight and passed through gradient concentration of alcohol for dehydration. Then, they were embedded in paraffin wax, and sections were then cut longitudinally and serially on glass slides. After heating the slides at 65 °C for 2 h, rehydration was performed using xylene I (20 min), xylene II (20 min) and a graded series of ethanol (100, 95, 80, and 75%), followed by washing in phosphate- buffered saline (PBS) three times for 5 min. Antigen retrieval was conducted using citric acid after heating to boiling in a microwave oven twice for 7 min. The sections were then blocked with 10 % goat serum for 0.5-1 h at 37 °C before incubation against the primary antibodies. DDX4 (1:200 dilution; Abcam) and BrdU (1:250 dilution; Abcam) at 4 °C overnight. After rewarming to room temperature for 1 h and washing three times for 5 min in PBS, the sections were incubated with secondary antibodies conjugated to fluorophores at 37 °C for 30 min. Then, the sections were washed in PBS 3 times and stained with DAPI (1:2000 dilution) for 15 min. Finally, the sections were mounted in 10 % glycerol in PBS and were then assessed using fluorescence microscopy.

### Immunohistochemistry

The steps for antigen retrieval were the same for immunofluorescence. Next, procedures were performed according to the instructions of the immunochemistry kit (ZSGB-BIO, Beijing, China). The sections were then incubated at 4 °C overnight with the following primary antibodies: SOD2 (1:200 dilution, Abcam) and 4-HNE (1:200 dilution, Abcam). Images were acquired for each section using a BX53F microscope (Olympus Corporation, Tokyo, Japan). The expression levels of related proteins were evaluated based on the mean optical density using Image Pro-Plus software 6.0 (IPP 6.0, Rockville, Maryland, USA).

### EdU assay

The effects of the gradient concentration of Res on OSC proliferation were analyzed by using an EdU incorporation assay kit (RiboBio, Guangzhou, China). OSCs were cultured in 24-well plates, treated with Res (0, 2, 20 and 100 μM) and incubated for 24 h. The following process of the EdU assay was based on the manufacturers’ instructions. The proportions of EdU-positive cells were calculated according to our previous studies [57].

### Cell cycle analysis

OSCs were treated as mentioned above. Cell cycle distribution was assessed by propidium iodide (PI) staining. OSCs were digested with 0.25 % trypsin, resuspended as single cells and washed in precooled PBS. Detached cells were fixed in 70 % ethanol at 4 °C for 24 h. After washing with PBS, the cells were stained with PI (50 μg/ml) and RNase A (10 mg/ml) at 37 °C for 30 min and subsequently analyzed by flow cytometry (Beckman Coulter, Brea, CA, USA).

### Cell apoptosis assay by flow cytometry

Apoptotic activity was analyzed using an Annexin V-FITC apoptosis detection kit (BD Pharmingen, San Diego, CA, USA). Cells were harvested and washed twice with ice-cold PBS and resuspended in 100 μl binding buffer. Then, 5 μl Annexin-V and 5 μl PI (2.5 μg/ml) were added, and the cells were gently vortexed and incubated for 15 min at RT (25 °C) while protected from light. Finally, 200 μl binding buffer was added to each sample tube. The percentage of apoptotic cells was analyzed by a flow cytometer (Beckman Coulter) within 1 h.

### Determination of ROS generation

OSCs were harvested and then treated with 500 μl DCFH-DA (ROS Assay Kit; Beyotime, Haimen, Jiangsu, China) in serum-free medium (1:1000 dilution). The cells were subsequently incubated in the dark for 20 min at 37 °C. The cells were then washed twice with serum-free medium. Finally, the samples were resuspended in 150 μl PBS and analyzed by flow cytometry (Beckman Coulter).

### Western blot analysis

OSCs treated with Res were collected, and proteins were extracted from the cells and quantified using Coomassie brilliant blue G250 according at standard curve. The proteins were separated using 10 % SDS-PAGE and then transferred to PVDF membranes. The membranes were blocked with 5 % nonfat milk in TBST for 1 h and were then incubated with diluted primary antibodies overnight at 4 °C. After rewarming for 1 h on the second day and washing for 3 times in TBST, the membranes were incubated with secondary antibodies (1:2000 dilution) at 37 °C for 1 h and washed with TBST. Protein bands were visualized using a Bio-Rad imaging system and Western Bright ECL and Peroxide (Advansta, San Francisco, CA, USA). All protein bands were imaged using a ChemiDoc TMXRS+system and Image Lab^TM^ software (Bio-Rad, Hercules, CA, USA). All antibodies were listed in Supplementary Table 1. Each experiment was repeated independently in triplicate.

### RNA extraction and qRT PCR

Total RNA was extracted from ovaries from 4 groups (Blank, Chemo, 30 Res and 100 Res) using RNAiso plus reagent (Takara, Nojihigashi, Japan). Samples consisting of 2 μg total RNA from each group were digested with RNase-free DNase I (Thermo Fisher Scientific) and then reverse transcribed into cDNA using Transcriptor reverse transcriptase (Takara, Japan). qRT-PCR analysis was performed in triplicate on a CFX96 real-time PCR system (Bio-Rad) at a final volume of 20 μl. Each reaction contained 10 μl SYBR Green Mix (Bio-Rad), 1 μl cDNA, 8 μl ddH_2_O, and 1 μl primer mix. The Gapdh gene was used as the housekeeping control. The qRT PCR primers are listed in Supplementary Table 2. The details of the PCR primers used in RT-PCR for mouse ovaries and OSCs are described by Wu [58].

### Statistical analysis

All experiments were independently replicated at least three times. One-way analysis of variance (one-way ANOVA) was performed, followed by post hoc tests using Dunnett’s and Turkey’s multiple comparisons. Baseline data are expressed as the means (±SD). A P-value of P < 0.05 was considered statistically significant. Statistical analysis of all data was performed using GraphPad Prism 7.0 (GraphPad Software, San Diego, CA, USA).

## SUPPLEMENTARY MATERIALS

Supplementary Tables
