# Correction for: Resveratrol alleviates chemotherapy-induced oogonial stem cell apoptosis and ovarian aging in mice

**DOI:** 10.18632/aging.102852

**Published:** 2020-02-11

**Authors:** Meng Wu, Lingwei Ma, Liru Xue, Wenlei Ye, Zhiyong Lu, Xiang Li, Yan Jin, Xian Qin, Dan Chen, Weicheng Tang, Yingying Chen, Zixin Hong, Jinjin Zhang, Aiyue Luo, Shixuan Wang

**Affiliations:** 1 Department of Obstetrics and Gynecology, Tongji Hospital, Tongji Medical College, Huazhong University of Science and Technology, Wuhan, Hubei 430030, China; 2 Hubei Key Laboratory of Embryonic Stem Cell Research, Tai-He Hospital, Hubei University of Medicine, Shiyan, Hubei 442000, China

**Keywords:** correction

**This article has been corrected:** The authors requested to replace Figure 4E and Figure 6. The mistakes of these figures are described below:

**Figure 4E:** The authors submitted wrong c-kit in panel E of Figure 4.

**Figure 6:** The order in panels D and E of Figure 6 was reversed. The H2O2 and the H2O2+Res group in the original Figure 6D of apoptotic analysis was reversed.

These corrections do not change any of the conclusions of the publication. The corrected Figure 4E and Figure 6 are provided below.

**Figure 4:**
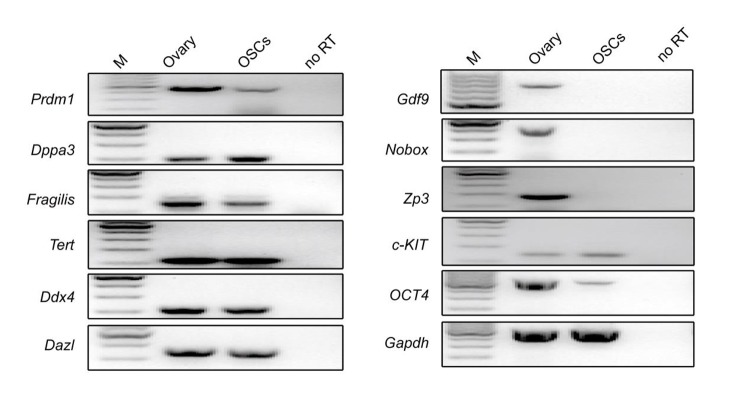
**Morphology and characteristics of OSCs. **(**E**) Reverse transcription PCR analysis for the expression profile of OSCs using ovarian tissue as a positive control. M: 100 bp DNA marker; No RT, PCR of RNA sample without reverse transcriptase.

**Figure 6:**
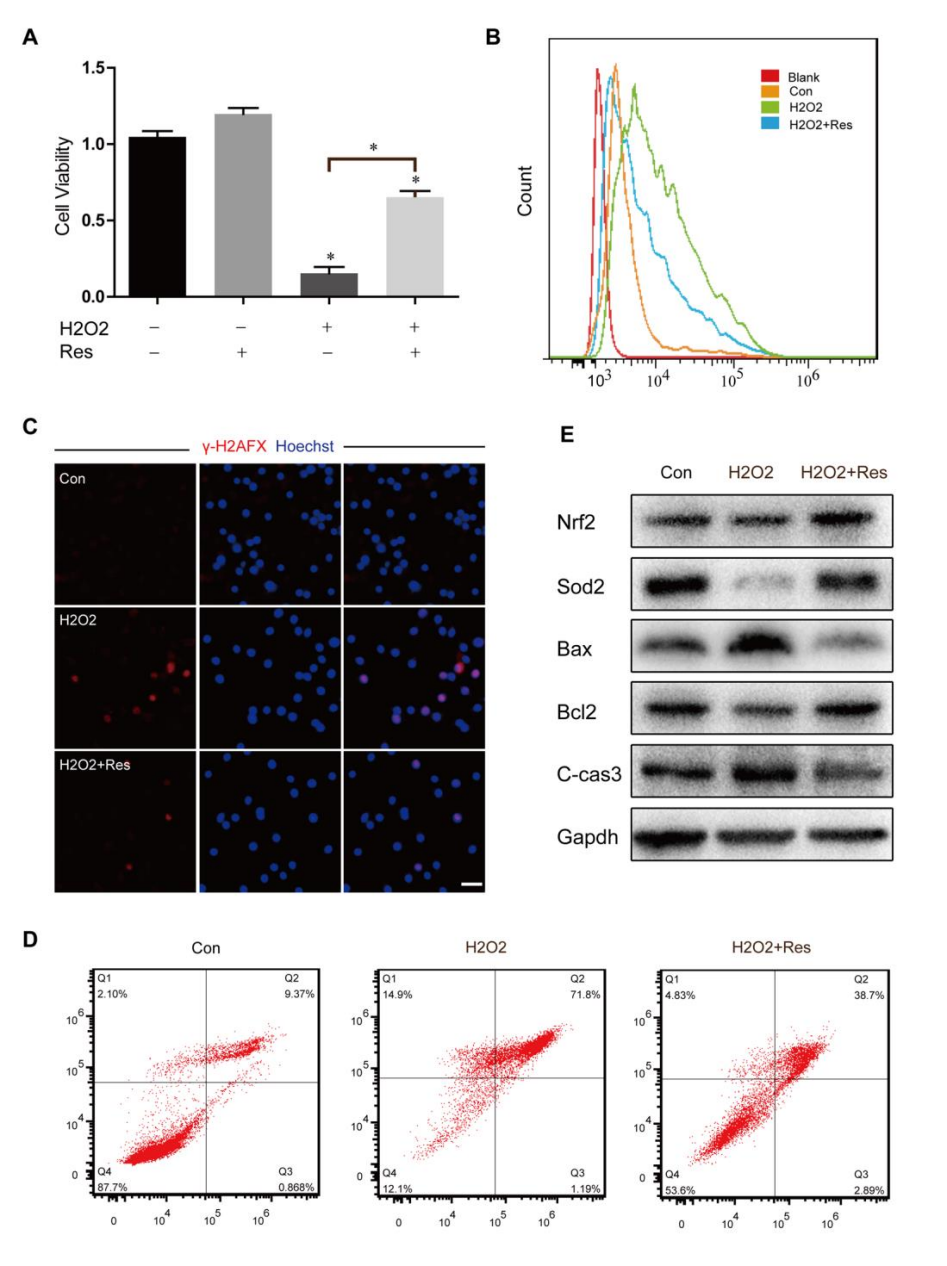
**Resveratrol attenuated H2O2‐induced cytotoxicity and oxidant stress injury in OSCs.** (**A**) CCK8 assay for treated OSCs;*p < 0.05. (**B**) Analysis of intracellular ROS by cell flow cytometry. (**C**) Immunofluorescence staining of γ‐H2AX and Hoechst. Scale bar: 50 μm. (**D**) The flow cytometry apoptotic analysis of treated OSCs. (**E**) Western blotting of related protein expression levels in treated OSCs.

Original article: Aging. 2019; 11:1030–1044.  https://doi.org/10.18632/aging.101808

