# Supplementary material for: Resveratrol alleviates chemotherapy-induced oogonial stem cell apoptosis and ovarian aging in mice
Source: Aging (Albany NY). 2019 Feb 14;11(3):1030–44. doi: 10.18632/aging.101808 (PMC6382418; doi:10.18632/aging.101808)
Supplement: Supplementary Tables [file aging-11-101808-s001.pdf]

SUPPLEMENTARY TABLES

Supplementaty Table 1. The antibodies information.

Antibody	Corporate Brand
DDX4	Abcam, Cambridge, MA, USA
Fragilis	Abcam
Oct4	Abcam
BrdU	Abcam
SOD2	Abcam
NTY	Abcam
4-HNE	Abcam
GAPDH	Abcam
Nrf2	Proteintech, Rosemont, USA
Bcl-2	Cell Signalling Technology, Danvers, MA, USA
Bax	Abclonal, Boston, MA, USA
c-caspase3	Servicebio, Wuhan, China

Supplementary Table 2. The qRT-PCR primers sequences used in the manuscript.

		Primer sequence, 5'-3'	
**Gene**	**Genbank ID**	**Forward**	**Reverse**
c-KIT	NM_021099z	CTGGTGGTTCAGAGTTCCATAGAC	TCAACGACCTTCCCGAAGGCACCA
Oct4	NM_013633	AGCTGCTGAAGCAGAAGAGG	GGTTCTCATTGTTGTCGGCT
Sox2	NM_011443	TAGAGCTAGACTCCGGGCGATGA	TTGCCTTAAACAAGACCACGAAA
Nanog	NM_028016	CAGGAGTTTGAGGGTAGCTC	CGGTTCATCATGGTACAGTC
Gdf9	NM_008110	TGCCTCCTTCCCTCATCTTG	CACTTCCCCCGCTCACACAG
Ddx4	NM_010029	ACCCAGTTTGGTCATTCAGTTCG	TTGTTCCTTTGATGGCATTCCTG
Gapdh	NM_008084	GTCCCGTAGACAAAATGGTGA	TGCATTGCTGACAATCTTGAG
